# Testing Associations Between Key Precarities and Adverse Outcomes in Older Adults Without Care Partners

**DOI:** 10.1093/geroni/igaf122.4020

**Published:** 2025-12-31

**Authors:** Brittany Jones-Cobb

**Affiliations:** University of Washington, Seattle, Washington, United States

## Abstract

Many older adults in the U.S. rely on care partners such as a spouse, child, kin, or other social network member to assist with daily needs, yet demographic trends are leaving more individuals without such vital support. This study builds on a scoping review that identified social isolation, future care planning (e.g., advance directives), self-efficacy, and engagement in preventive services as key precarities for older adults without care partners, and hospitalization and functional decline as potential adverse outcomes. Using longitudinal data from the Health and Retirement Study (2006–2020), hybrid mixed-effects logistic regression models with random intercepts estimated within- and between-person effects of these precarities on hospitalization and incident activities of daily living (ADL) dependency among respondents without care partners, controlling for age, sex, race, Hispanic ethnicity, and poverty status. For hospitalization, higher perceived constraints (OR = 1.27, 95% CI = 1.11–1.46) and lower average future care planning (OR = 0.77, 95% CI = 0.68–0.88) were associated with greater odds. For ADL dependency, higher perceived constraints (OR = 1.49, 95% CI = 1.14–1.94), greater social isolation (OR = 1.32, 95% CI = 1.04–1.68), and lower perceived mastery (OR = 0.63, 95% CI = 0.49–0.82) were associated with greater odds, with heightened risks for Hispanic respondents (OR = 3.27, 95% CI = 1.43–7.51) and those experiencing poverty (OR = 2.52, 95% CI = 1.45–4.37). Findings suggest interventions targeting self-efficacy, social isolation, and care planning may reduce risks of hospitalization and ADL dependency among older adults aging without care partners.

